# A novel method for histopathological analysis of oral squamous cell carcinoma: a pilot study using three-dimensional imaging to enhance local control

**DOI:** 10.3389/fonc.2025.1617778

**Published:** 2025-11-04

**Authors:** Adam Michcik, Rafał Pęksa, Łukasz Garbacewicz, Adam Polcyn, Piotr Choma, Barbara Wojciechowska, Barbara Drogoszewska

**Affiliations:** ^1^ Department of Maxillofacial Surgery, Faculty of Medicine, Medical University of Gdansk, Gdańsk, Poland; ^2^ Department of Maxillofacial Surgery, University Clinical Centre in Gdańsk, Gdańsk, Poland; ^3^ Department of Pathomorphology, Faculty of Medicine, Medical University of Gdansk, Gdańsk, Poland; ^4^ Department of Clinical Pathomorphology, University Clinical Centre in Gdańsk, Gdańsk, Poland

**Keywords:** oral squamous cell carcinoma, histopathological analysis, three-dimensional imaging, local control, 3D objects, virtual medicine

## Abstract

One factor that enhances the survival of patients with oral squamous cell carcinoma (OSCC) is improved local control. Many prognostic factors (PF) influence a patient’s prognosis, one of which is the correct histopathological excision margins. Standard histopathological analysis does not allow for the precise determination of the locations of individual hematoxylin and eosin (H&E) slides, which is clinically very important. In extensive tumors, the ability to precisely identify the sites with the narrowest microscopic excision is essential for accurately interpreting postoperative results and making proper clinical decisions. Therefore, a protocol has been developed to precisely localize individual H&E-stained tissue slides on virtual three-dimensional (3D) OSCC images. This approach provides a virtual map of the entire excised tumor, allowing accurate identification of areas with the narrowest histopathological margins (HM). As a result, if reoperation is necessary, it is possible to precisely determine where to widen the OSCC excision or plan adjuvant radiotherapy with greater accuracy. The proposed pilot study is based on the virtual three-dimensional OSCC model of the floor of the mouth cancer. Using 3D scanning technology, a user-friendly protocol has been created.

## Introduction

Among malignant tumors, oral squamous cell carcinoma ranks 16th worldwide (1) and is the most common malignant tumor of the oral cavity (2, 3). Persistent unfavorable outcomes, including a 5-year survival rate of 50% (4), remain unsatisfactory. The literature describes numerous prognostic factors (PF) that influence patient prognosis (5). Some of these are determined by the biological characteristics of oral squamous cell carcinoma (OSCC) (6–8) and nodal metastases (9, 10), which increase the risk of treatment failure. The depth of invasion (DOI), lymphovascular invasion (LVI), perineural invasion (PNI), and extranodal extension (ENE) (11–13) are of significant importance and directly influence the risk of recurrence. The worst pattern of invasion (WPOI) describes tissue infiltration by cancer cells at the tumor interface and is considered a key factor in histological grading systems, especially for OSCC (14). It is well known that when nodal metastases are present, regional control of the tumor is challenging, and the likelihood of nodal recurrence is increased (9). According to existing research, locoregional spread is the most common cause of treatment failure in patients with OSCC (15, 16). However, tumor local control is equally essential. To increase it, procedures should be performed with a safe macroscopic excision margin, defined as 1 cm of macroscopically unchanged tissue around the tumor (17, 18). The UK Royal College of Pathologists issued guidance on pathological margins for OSCC (19). A margin less than 1 mm is considered involved, less than 5 mm as close, and greater than 5 mm as clear. In available studies, we observe various cutoffs that have been identified as sufficient (20–22) and did not impair local tumor control. When planning OSCC excision with an appropriate margin, it is also necessary to consider how the processes involved in specimen preparation for histopathological analysis might affect the outcome. The influence of formalin fixation (FF) is important for the final measurement of the width of the microscopic margin width. Available publications confirm shrinkage occurring during FF (23–25).

Standard histopathological analysis of the resected OSCC includes cover-up and subsequent slicing according to a scheme recognized by the pathologist. During this process, the technician records the pathologist’s notes to aid in the later interpretation of the results. Then, the histopathological slides are prepared and stained using the standard hematoxylin and eosin (H&E) stain. Once the specimen is dissected, the ability to visualize the tumor and to connect the histopathological findings to it is lost, as well as the capacity to accurately identify which section of a given margin is affected by a specific H&E slide. This is especially critical in cases of irregular tumor shape and long margins. In such situations, the ability to retrospectively locate a specific area on the sample is essential. This underscores the need to explore new solutions to improve the accuracy of H&E slide locations and enable a more precise assessment of OSCC margins. The proposed pilot study assumed that three-dimensional (3D) scanning technology would create a virtual, interactive histopathological examination result that accurately and precisely determined each point of the excised and mapped cancer. This may improve the accuracy of histopathological slide analysis and enable archiving as well as interactive retrospective study analysis. The use of structured light in the histological analysis of OSCC is a novelty and opens up new, previously unattainable possibilities for digitizing this stage of patient oral cancer treatment.

## Methods of the pilot study

The presented methodology is based on over 100 scans of resected OSCCs and the virtual mapping method. This helped eliminate errors that occurred during the initial phase of the process.

### Scanning protocol

**Figure 1:**
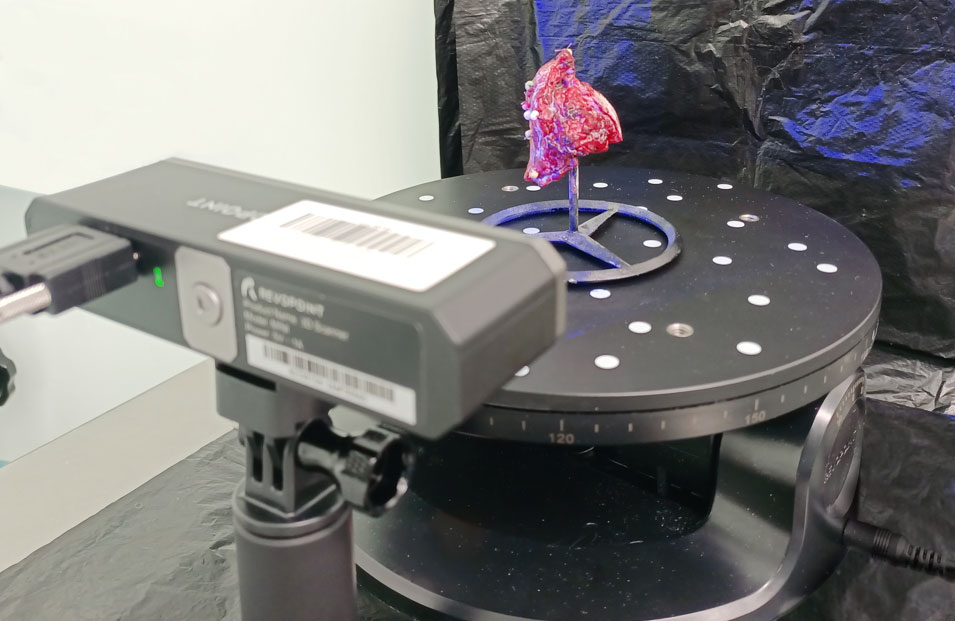
Tripod with tongue cancer model placed on a rotating table.

The excised OSCC with a margin of macroscopically unchanged tissue was marked in the operating room with beads to facilitate orientation in the subsequent 3D virtual object. Following the procedure, it was placed in formalin. After 24 to 48 h post-FF, the tumor was scanned according to our protocol in the pathology laboratory (26). After formalin fixation and drying, the tumor was placed on a specially constructed tripod (**Figure 1**).

**Figure 2:**
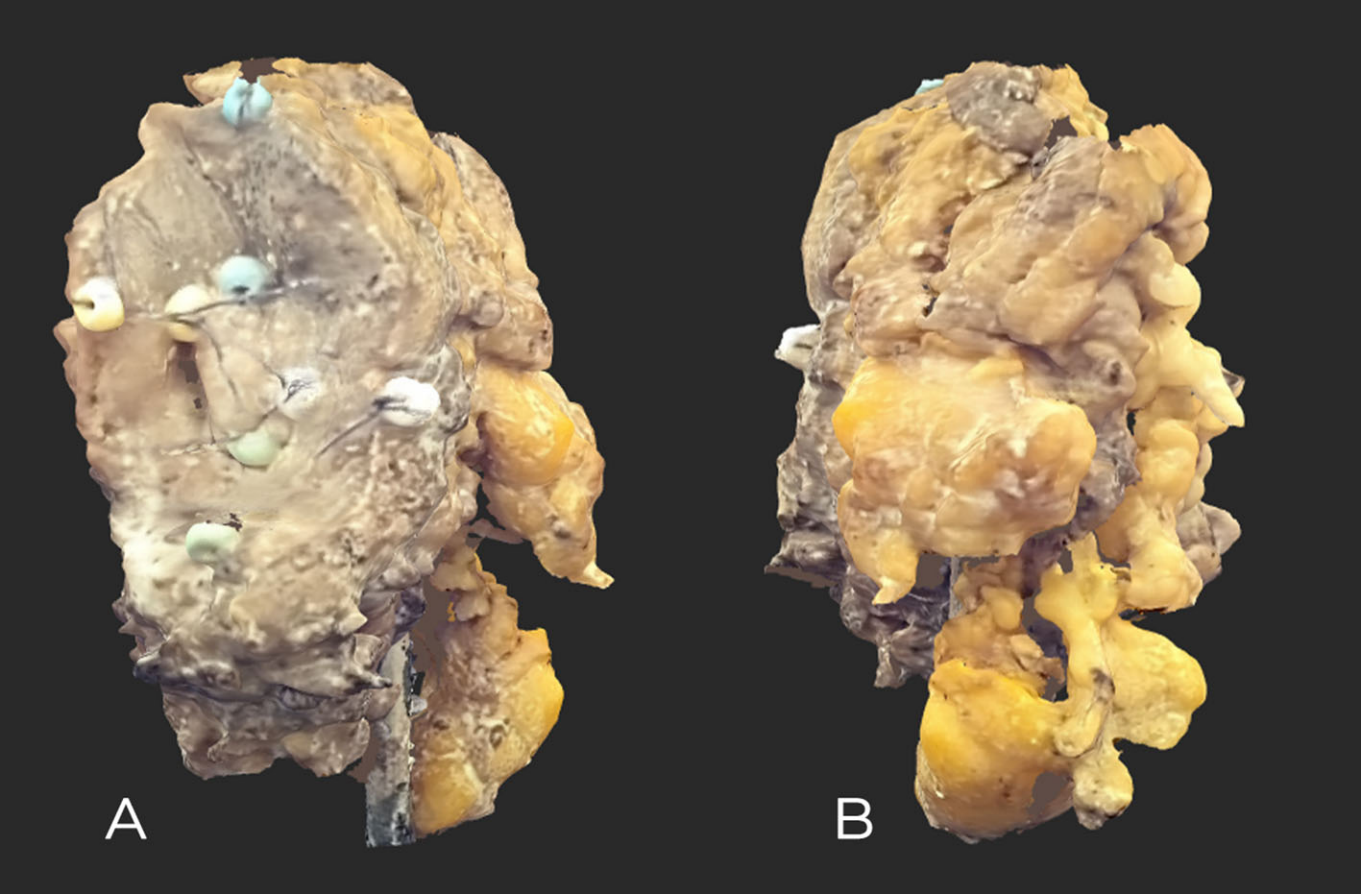
High-resolution maxilla tumor scan after FF. **(A)** Front view showing slight ulceration and sewn-on markers. **(B)** Rear view; Bichat’s buccal fat is visible.

The tumor was then contrasted with a matte spray designed for Aesub Blue 3D scanners (composition: propane, ethanol, tricyclodecane, hydrocarbons, C6–C7, iso-alkanes, cyclics, < 5% *n*-hexane, *n*-hexane) (27). Next, scanning was performed using a 3D scanner: Revopoint MINI—Dual-Axis Turntable Package (Shenzhen, China), adapted to scan small objects (minimum 10 mm × 10 mm × 10 mm) with an accuracy of 0.02 mm at 10 frames per second and a point distance of up to 0.05 mm. The scanner was selected for its exceptional resolution and resistance to ambient lighting. Finally, the acquired virtual models in STL format (triangulated surface geometry in three-dimensional space) were processed using Revo Scan 5 (https://global.revopoint3d.com), which included removing environmental contaminants and enhancing the tumor surface detail. The processed 3D images were saved as OBJ files, and the models’ textures were saved as JPG files. The total time to complete the described process was approximately 10 min (**Figure 2**).

### Image annotation

The image labeling process was conducted using Blender 4.1.1, an open-source application (www.blender.org, accessed on 25 September 2023). This software was chosen for its accessibility, free availability, and user-friendly interface. Originally developed as a 3D modeling tool for digital creators, its capabilities have expanded over time to include film editing and motion capture. The application allowed users to add annotations directly onto the surfaces of 3D objects and create markers with notes.

### Histopathological processing of the floor of the mouth cancer specimen with parallel annotation on the virtual OSCC object

#### Stage I: Covering up the specimen

**Figure 3:**
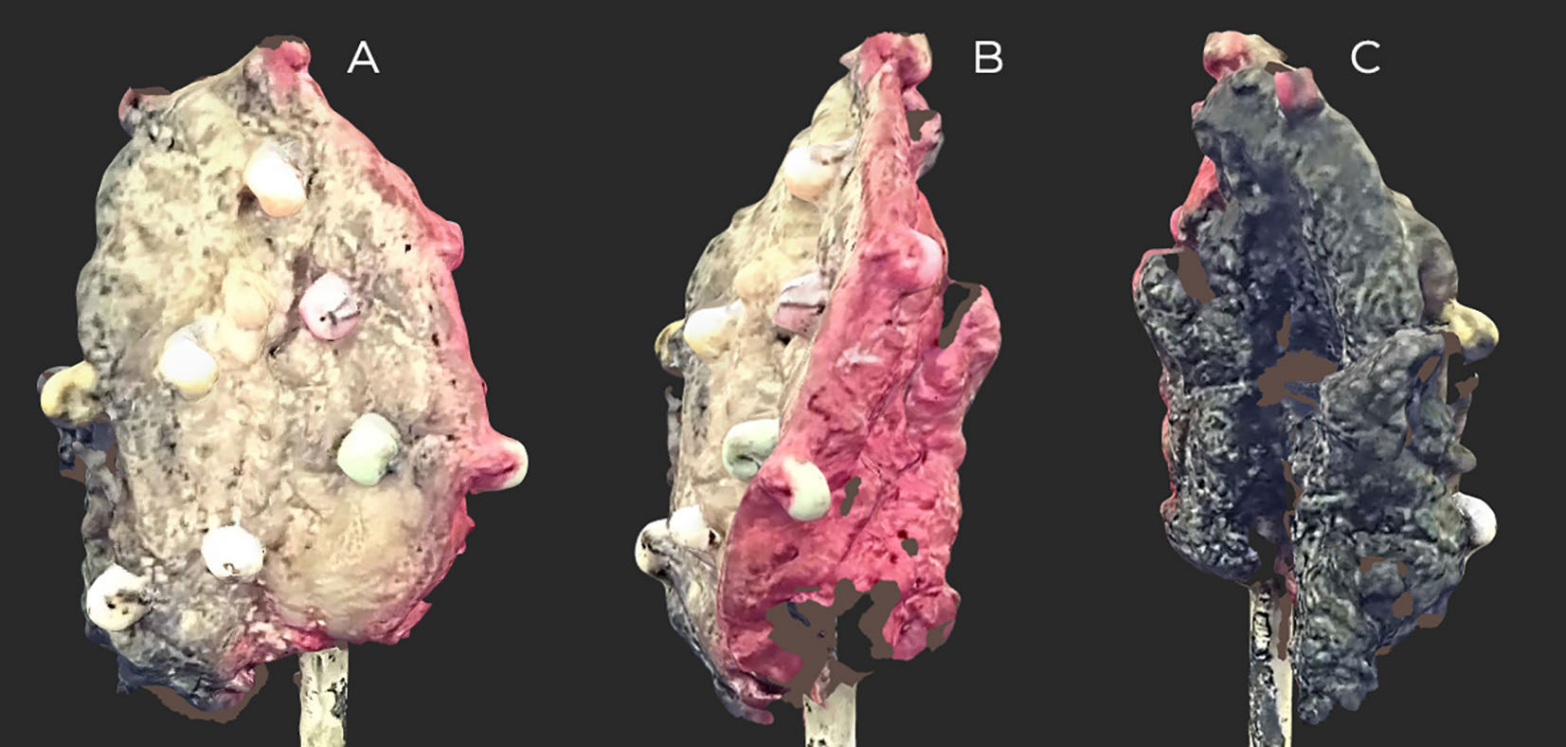
Scan of the inked floor of the mouth (FOM) specimen before slicing. On the black-inscribed specimen, small areas are invisible to the scanner. Inked: side and back, red; front and middle, black. **(A)** Front view. **(B**, **C)** Side views.

In the first stage of the pathological protocol, the specimen was inked (**Figure 3**).

#### Stage II

**Figure 4:**
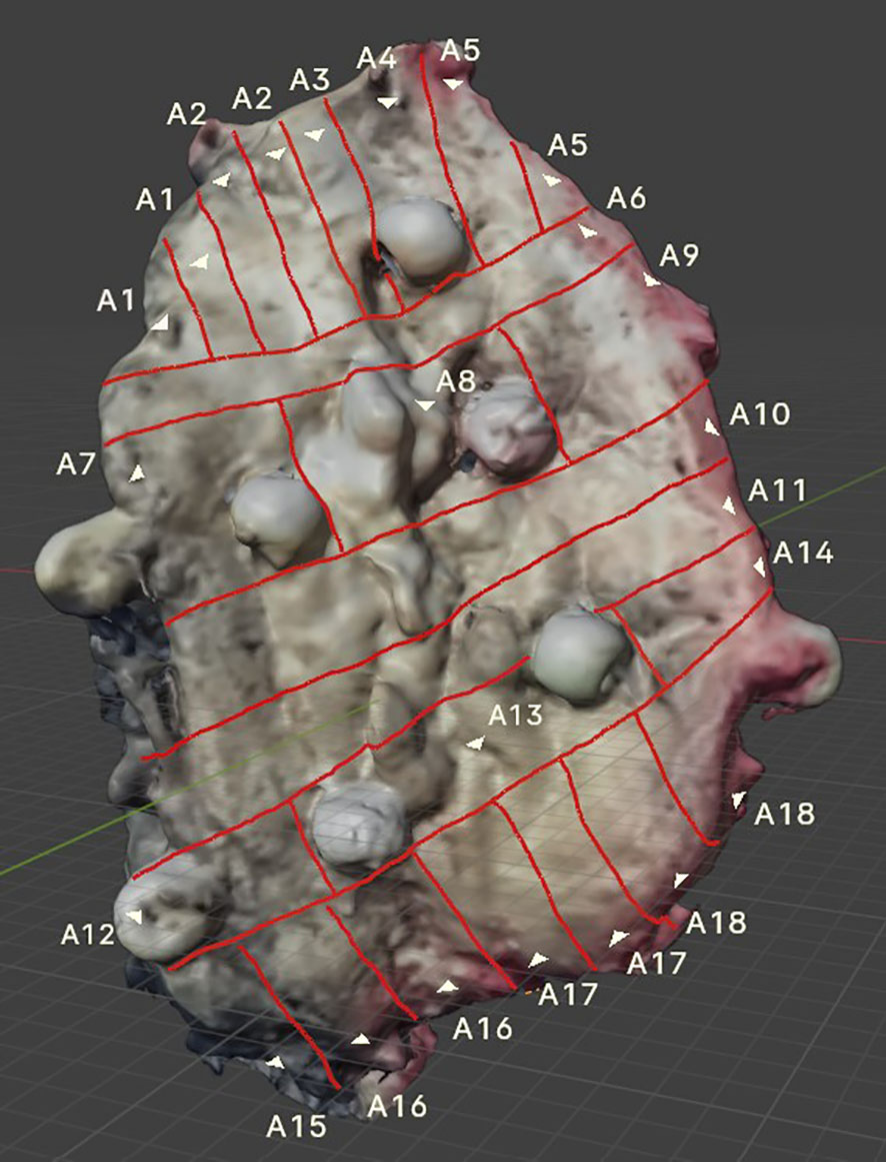
3D FOM cancer scan showing visible incision lines applied to the tumor surface, created in parallel with the incisions made on the actual specimen. Sewn-on markers enhance the accuracy of tumor mapping.

**Figure 5:**
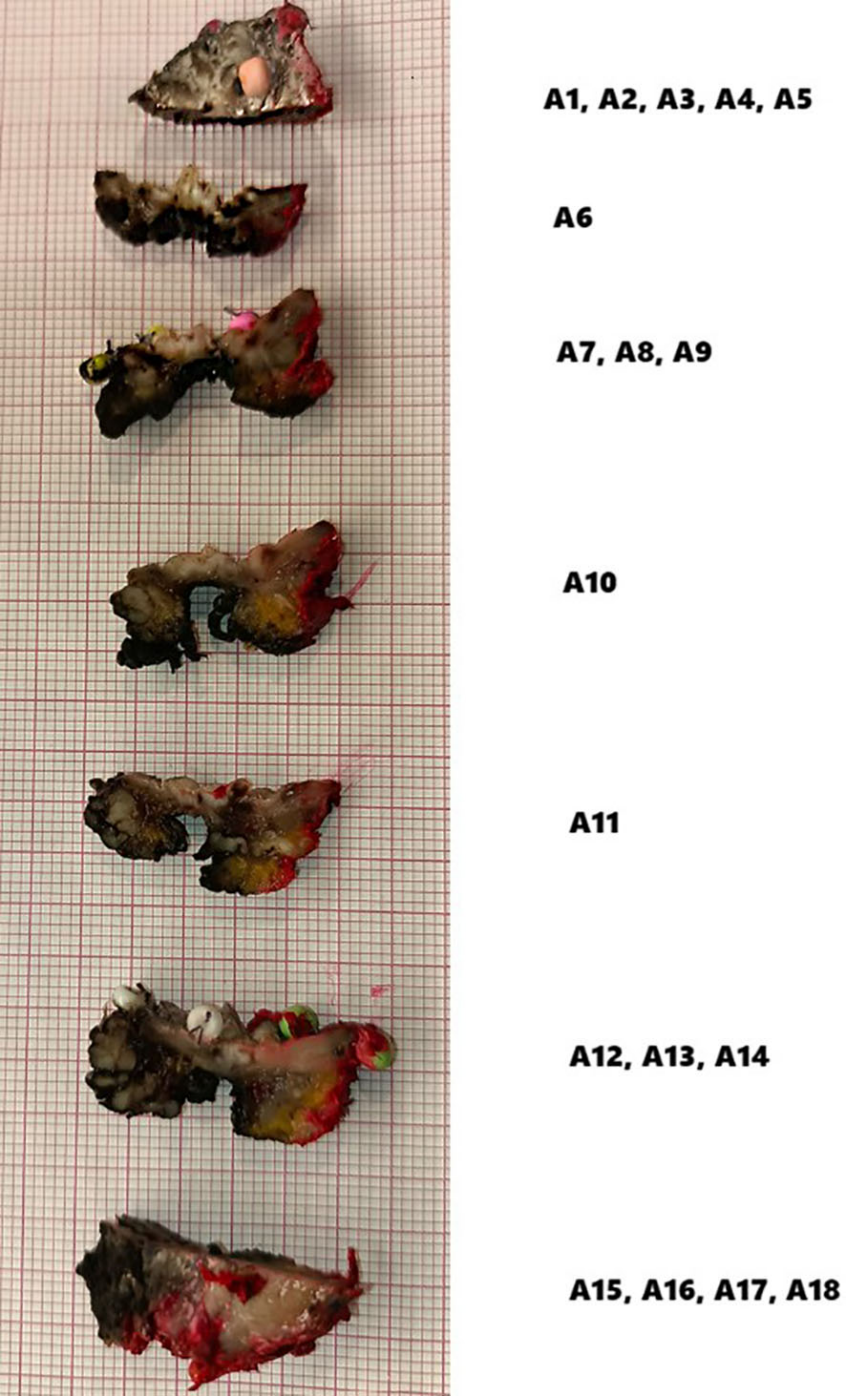
Photo of the same sliced FOM cancer specimen.

After FF, the specimen was sliced while simultaneously mapping the tumor on the scan. The visible lines correspond to the actual lines cut on the specimen by the pathologist (**Figures 4**, **5**).

### Histopathological analysis

**Figure 6:**
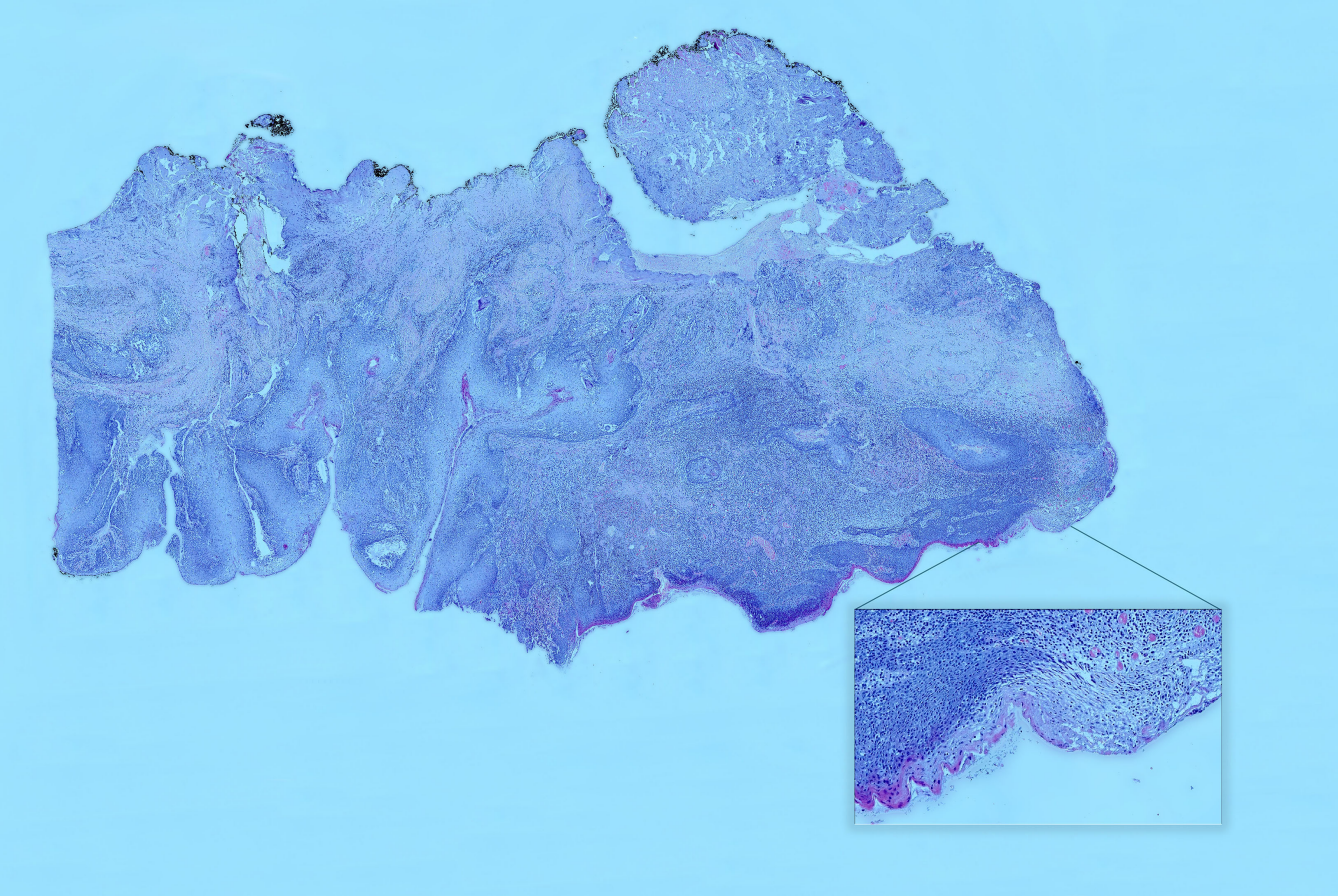
Hematoxylin and eosin (H&E) slice scan No. A3. A high-resolution scan showing the entire margin. A magnified view of a specimen fragment displays detailed images in an interactive histopathological report. In the interactive version, users can view H&E slide scans of specific areas selected on a virtual OSCC, providing easy and unlimited access to archived H&E images.

According to the presented numbering (A1–A18), H&E-stained tissue slides were collected (**Figure 6**).

## Results

**Figure 7:**
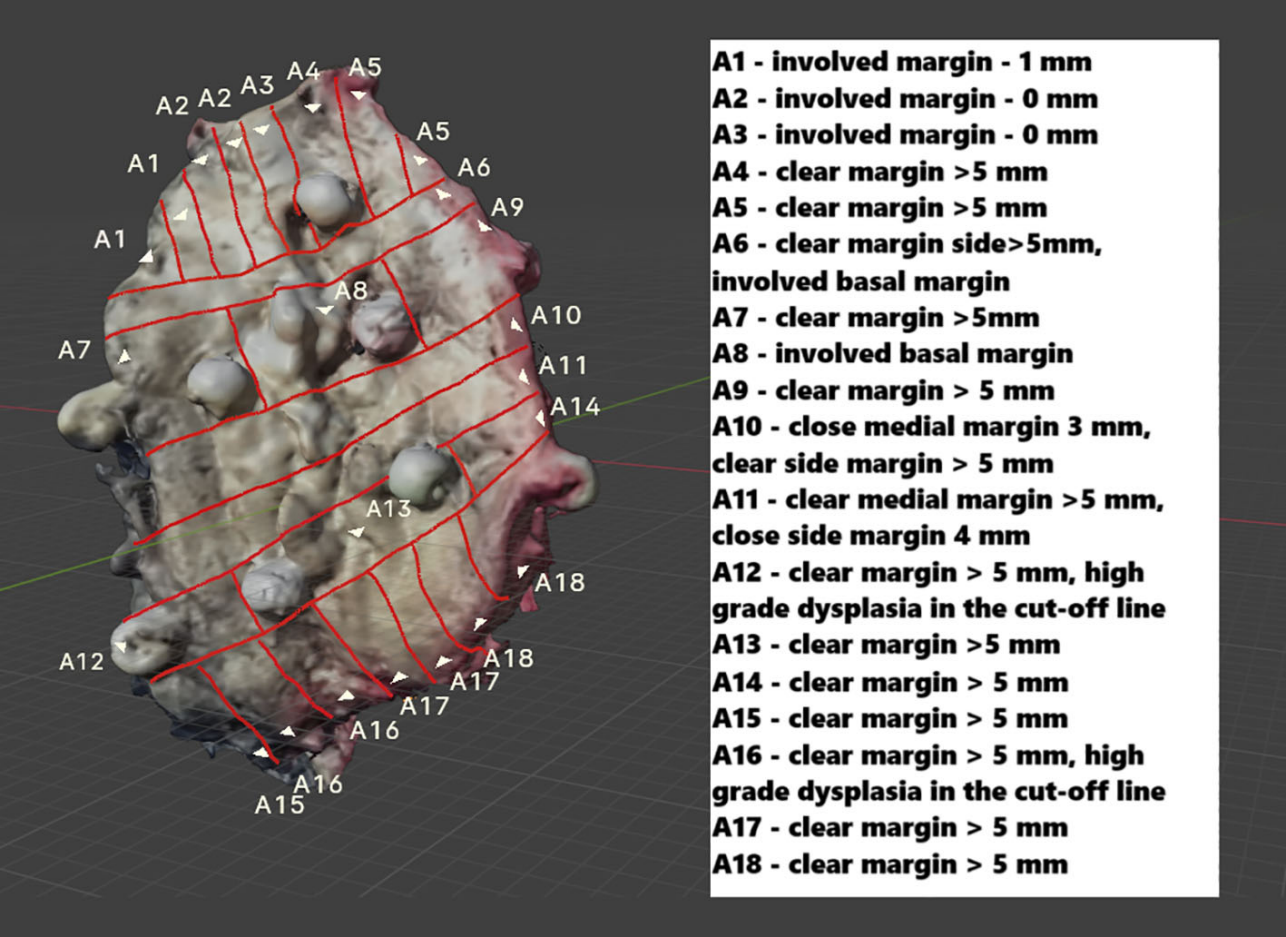
The mapped FOM scan includes a description of all evaluated H&E slides, enabling precise identification of areas with the narrowest margin. In the draft version, details about individual H&E slides can be provided as a legend (as shown earlier) or directly attached to a virtual object (expanded through cursor selection). Additionally, in the interactive version, users can upload H&E slide scans after selecting a specific area on a particular slide.

After the pathologist evaluated the received H&E slides, the results were recorded in a description of the virtually mapped tumor, numbered accordingly. Additionally, to assist with orientation, the tumor’s margins are described, and annotations have been added to support retrospective analysis (**Figure 7**).

**Figure 8:**
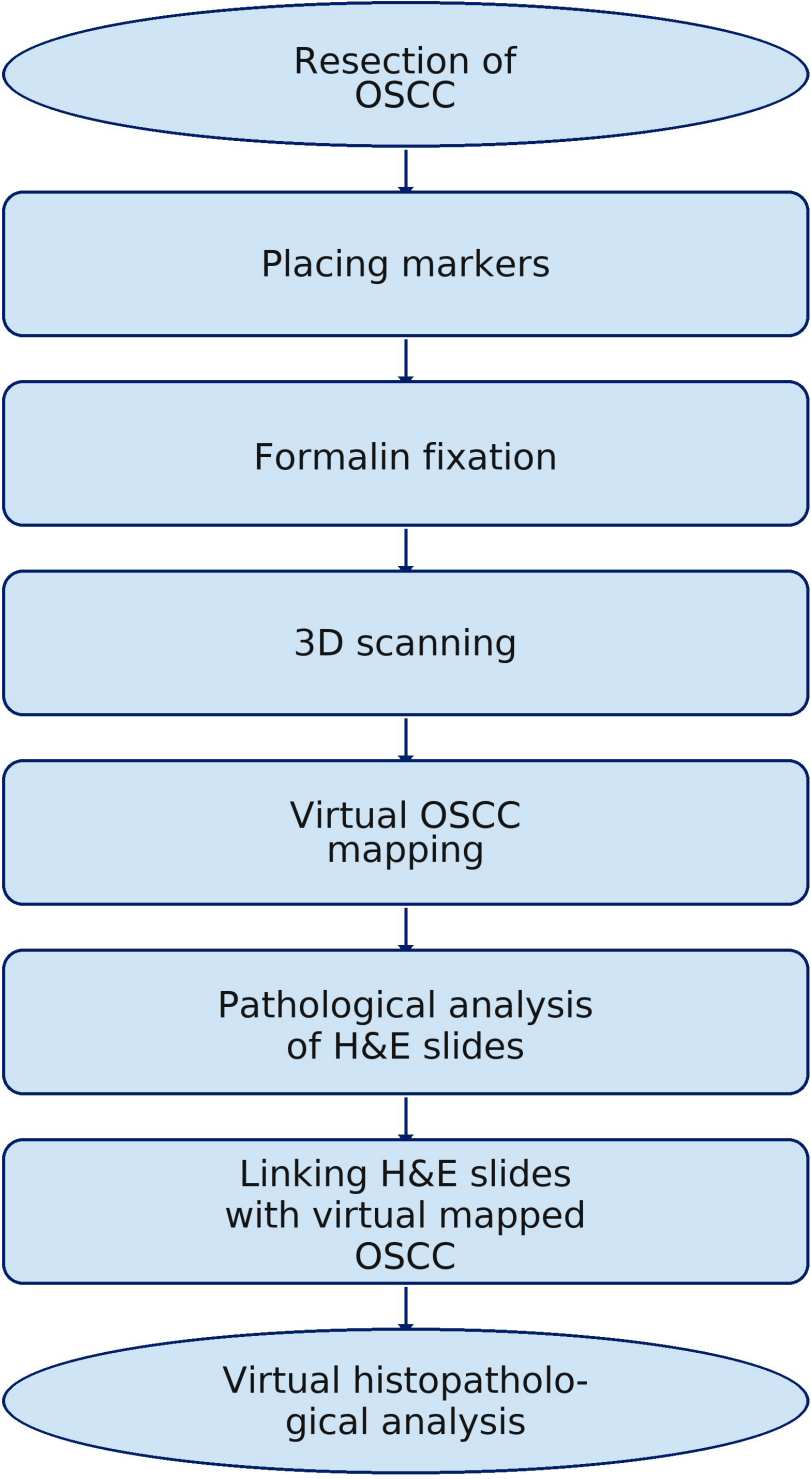
Workflow diagram of virtual histopathological analysis.

For simplicity and accessibility, the archived OSCC with histopathological margin (HM) results, initially saved as a.obj file (Blender 4.1.1), has been converted to a Portable Document Format (PDF) that allows interactive highlighting of specific areas corresponding to H&E slide numbers. While support for virtual objects in Blender 4.1.1 offers great editing options, it requires additional learning, which can be challenging. To make the file easier and more shareable, it was converted into a PDF that can be viewed on any computer with basic software. This significantly enhances the accessibility of virtual results, which is especially important during oncology team meetings or when sharing images with other centers. Consequently, there is greater potential for information flow among the surgeon, the pathologist, and, subsequently, the oncologist. This improved communication fosters a more integrated approach to patient care, ultimately contributing to more informed clinical decision-making (**Figure 8**, **Video**).

## Discussion

Using 3D scanning technology allowed modifications to the OSCC histopathological examination process. By archiving spatial, mapped 3D objects and creating an interactive map, we have gained previously unavailable opportunities for more precise interpretation of histopathological results. Currently, the locations corresponding to a specific H&E slide can be identified with high precision, even along long margins or within complex tumor shapes. This can help guide postoperative decisions, such as planning reoperations or adjuvant radiotherapy. The proposed method has the potential to improve local control of OSCC by more precisely identifying the narrowest areas of the microscopic margin. A postoperative clinical challenge faced by all surgeons treating OSCC is accurately interpreting histopathological results, including precisely determining the width of the HM along its entire length. In standard histopathological analysis, the result obtained is the H&E slide with the narrowest margin. In this case, it is not possible to identify which part of the margin the result pertains to. Furthermore, after cutting the specimen, the pathologist permanently loses the overall view and the ability to accurately identify the location of a specific slice later on. This makes it impossible to track the actual changes in the HM’s width along its entire length. Consequently, in some clinical cases, especially those involving primary extensive tumors, histopathological results are often difficult to interpret and can lead to incorrect conclusions. Making further therapeutic decisions based on standard histopathological analysis is often challenging and depends primarily on clinical experience.

It is well known that histological differentiation (28), DOI, LVI, PNI, a diffuse pattern of invasion (29), and pT stage (30) affect the local recurrence (LR). However, patients with close HM generally have a poor prognosis (31). Therefore, the postoperative therapeutic decision on eligibility for reoperation, observation, or adjuvant radiotherapy must be made based on an analysis of all the above factors. Studies indicate that margin status is one of the most important factors influencing local cancer control (31–33). Regardless of the factors mentioned, the margin status determines the radicalness (34). Therefore, increasing the detail of histopathological results, including identifying the narrowest points of the HM or detecting epithelial dysplasia, will improve the accuracy of interpretation. In the absence of the remaining PFs, the decision to reoperate will be based on the width of the HM. Precisely locating the site with the narrowest HM enables a more accurate, targeted reoperation. This is especially important in cases of extensive primary resections or near critical organs, where limiting the extent of surgical excision is necessary. Additionally, in radiotherapy planning, the oncologist can more precisely identify where to deliver the highest dose of radiation using a virtual map of the area. This approach will help reduce radiation-related complications, including osteoradionecrosis. The primary premise behind the proposed methodology is its potential to improve local control of OSCC through virtual tumor mapping.

A personalized approach to patients, digitalization of medicine, and the use of modern imaging techniques present new challenges and opportunities for improving the effectiveness of cancer treatment. The continuous advancement of precision in various areas of oncological surgery, including robotic surgery and virtual radiotherapy planning, requires updating the standard histopathological procedure. This procedure should align with and reflect the modern technologies used at other stages of patient care in OSCC.

The latest research on OSCC histopathological evaluation focuses on artificial intelligence (AI), which, through the use of specific algorithms, is expected to assist in histopathological diagnosis of OSCC (35–37). Using algorithms to quantify lesions, such as on the HALO platform, enables tissue segmentation with AI assistance (38). Despite the potential of AI in OSCC histopathological assessment, this technique does not allow the determination of the location of histopathological slides on the resected tumor. AI assists the pathologist in accurately evaluating the specimen. Further work is needed to integrate the proposed method with AI’s evolving capabilities. Currently, the technique requires manual processing and mapping on a virtual OSCC. In the future, by leveraging AI capabilities, specific stages of the proposed method can be automated, significantly speeding up and simplifying the procedure.

The ability to virtually combine individual patient treatment stages, including the surgical phase, with oncological therapy creates new opportunities for personalized treatment. Transferring a virtual, annotated 3D tumor model for planning adjuvant radiotherapy—allowing for precise identification of the narrowest or involved margins (HM)—will enhance the accuracy and likely the effectiveness of treatment.

However, the proposed methodology has limitations. It requires proper equipment and training for surgical and pathological teams, including scanning procedures and the use of appropriate software. Additionally, large tumors with highly irregular surfaces and multiple arcades may pose challenges for virtual imaging. Nevertheless, the advantages clearly outweigh the procedure’s inconveniences, which take no more than 10 min. We hope the proposed pilot study will establish a standard for OSCC histopathological analysis and contribute to improved local tumor control.

## Conclusion

In summary, the protocol for the virtual presentation of histopathological results of resected OSCC, proposed in our pilot study, significantly improves the accuracy of interpretation and the planning of further treatment steps. Using modern, noninvasive virtual imaging technologies, these methods can be easily and consistently integrated into our daily clinical practice. Additionally, the capacity to archive data that would otherwise be permanently lost in traditional histopathological assessments allows for retrospective analysis of specimens during oncology team meetings or in cases of late local OSCC recurrence. Due to its simplicity and reproducibility, the protocol has the potential to become a routine part of OSCC treatment protocols and training programs.

## Data Availability

The original contributions presented in the study are included in the article/**Supplementary Material**. Further inquiries can be directed to the corresponding author.

## Supplementary material

Video Interactive PDF with margin widths and the ability to view H&E slides.
